# Engaging Direct Care Providers in the Implementation of Montessori Programming for Dementia

**DOI:** 10.1093/geroni/igab046.578

**Published:** 2021-12-17

**Authors:** Natalie Douglas

**Affiliations:** Central Michigan University, Mt Pleasant, Michigan, United States

## Abstract

There is a need to engage direct care providers such as certified nursing assistants (CNAs) explicitly in efforts to implement innovative programming in long-term care environments. This presentation will outline engagement strategies that supported the implementation of Montessori programming in a community of 20 individuals living with severe dementia. Examples about positioning the CNA at the center of decision making, negotiating and building trust, cultivating opportunities for mutual consultation, creating spaces for new ideas to emerge, and synthesizing diverse perspectives will be highlighted. Although the program achieved positive outcomes on a number of measures including decreased responsive behaviors from people living with dementia, decreased negative qualities of relationships between CNAs and persons with dementia, and increased positive qualities of relationships between CNAs and persons with dementia, this presentation will focus on the “how” of engagement between the project team and the CNAs by highlighting qualitative data.

